# Docetaxel extravasation results in significantly delayed and relapsed skin injury: A case report

**DOI:** 10.3892/ol.2014.1921

**Published:** 2014-02-27

**Authors:** PEI-HUNG CHANG, MEI-TI WANG, YI-HUA CHEN, YU-YING CHEN, CHENG-HSU WANG

**Affiliations:** 1 Division of Hemato-Oncology, Department of Internal Medicine, Chang Gung Memorial Hospital, Keelung 20445, Taiwan, R.O.C.; 2 Department of Pharmacy, Chang Gung Memorial Hospital, Keelung 20445, Taiwan, R.O.C.; 3 Cancer Center, Chang Gung Memorial Hospital, Keelung 20445, Taiwan, R.O.C.

**Keywords:** docetaxel, chemotherapy, extravasation

## Abstract

Chemotherapeutic agents can result in extravasation, which is considered to be a serious complication. The increasing number of exposures to different cytotoxic agents experienced by each patient may enhance the prevalence of this complication. Docetaxel is widely used in the treatment of numerous solid tumors. Thus, the current report presents the case of a breast cancer patient who developed a significantly delayed skin reaction one day after docetaxel extravasation, a rare skin manifestation, and relapsed one week subsequently. This unusual clinical presentation is an indicator that practitioners are required to carefully monitor the patient for further cutaneous reactions in the weeks following extravasation to observe any additional adverse reactions.

## Introduction

Extravasation is the leakage or direct infiltration of a chemotherapeutic agent from a vessel into the surrounding tissues (1) and has been reported to occur in 0.1–6.5% of chemotherapy infusions (2). Docetaxel is an antineoplastic agent widely used in the treatment of numerous solid tumors, including breast, lung, ovarian and prostate carcinomas (3). The well-known adverse effects of docetaxel include neutropenia, fluid retention, neuropathy, hypersensitivity reaction, alopecia, mucositis or nail changes (4). However, extravasation of docetaxel has been reported to cause significant local tissue injury, despite the fact that it was commonly classified as a non-vesicant chemotherapeutic agent (5). This report presents the case of a breast cancer patient that exhibited a significantly delayed skin reaction one day after extravasation and relapsed one week following docetaxel extravasation. The patient provided written informed consent.

## Case report

**Figure 1:**
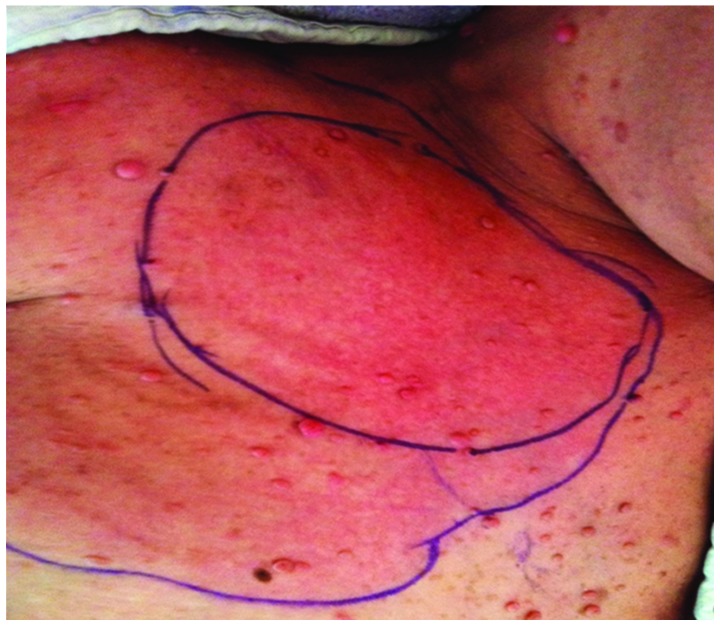
Marked swelling, erythema and pruritis involving the left chest wall one day after docetaxel extravasation.

**Figure 2:**
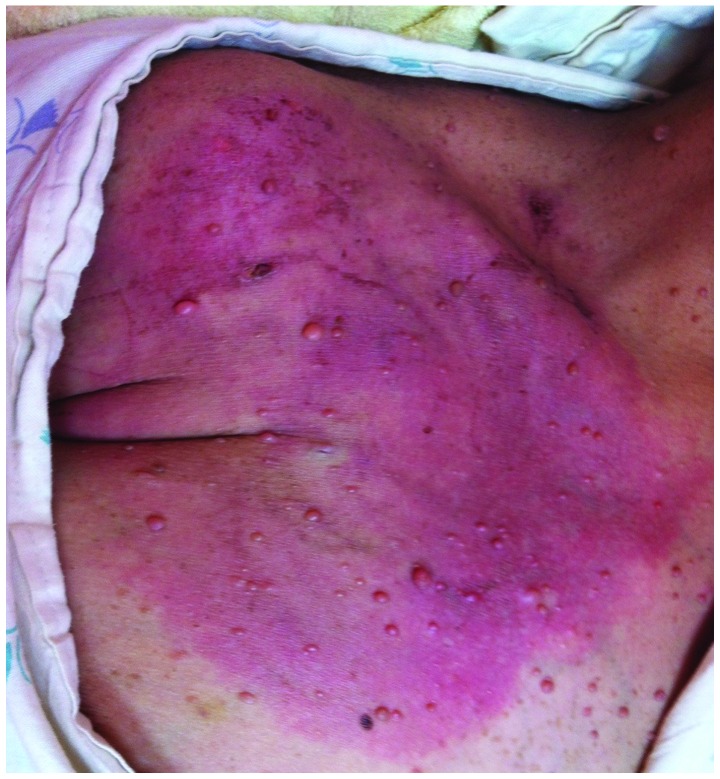
Erythema relapsed one week after docetaxel extravasation.

A 47-year-old female diagnosed with pathological stage IC triple negative breast infiltrative ductal carcinoma received a modified radical mastectomy of the left breast in October 2012. The patient was admitted to the Chang Gung Memorial Hospital (Keelung, Taiwan) where they received the first cycle of docetaxel in March 2013, in an adjuvant setting. The chemotherapy was administered via a portacath inserted into the left subclavian vein at a dose of 75 mg/m^2^ docetaxel diluted in 250 ml of 0.9% saline. Once ~250 ml was administered, the patient noticed a wet sensation and swelling in a small area around the cannula due to an accidental extravasation; the infusion was immediately discontinued. An aspiration of docetaxel was not possible, therefore, the cannula line was withdrawn. No immediate abnormalities were noted at the infiltration site and local cooling was initiated. However, on the following day the symptoms worsened with marked swelling, erythema and pruritus of the right chest wall, spanning an area of ~17×11 cm (Fig. 1). The range of motion of the left shoulder was limited by the tenderness and edema. The patient was treated with subcutaneous methylprednisolone around the site of extravasation; in addition, 5 mg intravenous dexamethasone and 5 mg chlorpheniramine were administered two times per day for three days. The symptoms of pain and swelling improved thereafter. Unfortunately, the erythema relapsed and progressed to 18×15 cm in size one week following extravasation (Fig. 2). Topical steroid ointment and systemic dexamethasone was prescribed and the erythema gradually improved. Three weeks after extravasation, desquamation of the affected skin occurred and slight erythema was observed. There was only a mild hyperpigmentation and the range of motion returned to normal in the left shoulder. Furthermore, an angiography examination of the portacath revealed no abnormalities. The patient received an additional three cycles of docetaxel infusion without experiencing further skin reactions.

## Discussion

Extravasation has been reported to occur in 0.1–6.5% of chemotherapy infusions (2). The increasing number of different cytotoxic agents that patients are exposed to may increase the prevalence of this complication. There are various chemotherapeutic agents, which may be classified as either irritants or vesicants depending on the reaction that is produced following extravasation onto the skin (3). Irritant agents cause pain at the injection site or along the vein, and may or may not result in an inflammatory reaction. Vesicant agents are more significant as they may induce a wide spectrum of lesions varying from mild erythema, swelling, and formation of blisters to tissue destruction, including ulceration and necrosis (3). Paclitaxel is a member of the taxoid family that has been recognized as a clear vesicant agent (4). By contrast, docetaxel, also a member of this family, is increasingly used to treat a wider spectrum of solid tumors and has been widely classified as an irritant; however, its vesicant potential should not be ignored (3,5).

The early manifestations of docetaxel extravasation may be subtle. Furthermore, the clinical symptoms may immediately appear following a leakage or may be delayed for weeks. Raley *et al* (3) reported that a patient developed clinical symptoms of extravasation, including erythema, pain and blisters, six days after a docetaxel extravasation. By contrast, our patient did not present with symptoms or signs of extravasation immediately; the symptoms developed after one day. The patients’ symptoms markedly improved following medical management, however, the skin erythema relapsed and progressed one week later. The differential diagnoses of skin lesions include recall dermatitis and fixed drug eruptions. The combination of persistent skin lesions following an extravasation as well as aggravation of the symptoms within seven days are unlikely to result in a diagnosis of recall dermatitis (6). As the skin lesions developed only at the extravasation site and there was no previous history of docetaxel treatment, an extravasation reaction was the favored diagnosis, rather than a fixed drug eruption. Although there is no consensus regarding the optimum treatment for extravasation injuries, cooling with ice, and the administration of topical steroids and antibiotics appear to provide good results (7). In addition, anti-inflammatory agents may be administered to relieve pain; however, to the best of our knowledge, plastic surgery has not been used to treat docetaxel extravasation in the published literature to date.

In conclusion, this report presented a rare case of a delayed skin reaction and a relapse of symptoms following a docetaxel extravasation injury. This unusual clinical presentation indicates that practitioners should carefully monitor additional skin reactions in the weeks following an extravasation to observe whether delayed or relapsed reactions occur. In addition, clinical practitioners should be more aware of this potential complication so as to better diagnose and promptly treat the symptoms, and avoid sequelae (8); a better outcome would be to avoid injury by improving the use of venipunctures and infusions. Patients should also be made aware of the possibility of an extravasation occurring, so that it may be reported promptly to minimize the extravasation-related symptoms.
